# Granola consumption with multiple prebiotics in Japanese participants increases *Bifidobacterium* abundance and improves stress and subjective sleepiness

**DOI:** 10.3389/fnut.2025.1551313

**Published:** 2025-03-20

**Authors:** Hiroyuki Sasaki, Hirofumi Masutomi, Shuji Nakamura, Chiemi Tanigawa, Yufei Cui, Katsuyuki Ishihara, Masashi Yanagisawa, Toshio Kokubo

**Affiliations:** ^1^Research & Development Division, Calbee, Inc., Utsunomiya, Japan; ^2^Sleep is the Ultimate Intelligent Mechanism In Nature (S’UIMIN) Inc., Tokyo, Japan; ^3^International Institute for Integrative Sleep Medicine (WPI-IIIS), University of Tsukuba, Tsukuba, Japan; ^4^The Department of Molecular Genetics, University of Texas Southwestern Medical Center, Dallas, TX, United States

**Keywords:** gut–brain axis, stress, sleep disorders, microbiota, prebiotics, granola

## Abstract

**Background:**

Sleep is essential for physical and mental health. However, stress-related sleep disorders are common in Japan, and the gut–brain axis may play a role in sleep and stress management. This study investigated whether the consumption of granola containing multiple prebiotic ingredients could alleviate stress and improve insomnia in adults with stress-related sleep problems, regardless of individual differences in the gut microbiota. Additionally, we aimed to investigate the relationship between changes in gut microbiota and the observed improvements.

**Method:**

A single-arm uncontrolled trial was conducted with 27 adults with high stress levels and sleep disturbance. The participants consumed 50 g of prebiotics-containing granola daily for 8 weeks. Subjective sleep quality was assessed using the Athens Insomnia Scale, Epworth Sleep Scale, and Oguri-Shirakawa-Azumi Sleep Inventory-Middle-aged and Aged version (OSA-MA). Stress levels were assessed by administering the Brief Job Stress Questionnaire and Profile of Mood States 2nd edition (POMS2). Gut microbiota composition was analyzed using 16S rDNA sequencing.

**Results:**

After 8 weeks, subjective insomnia scores and sleep onset and maintenance improved significantly, whereas the stress and mood disturbance scores decreased significantly. Gut microbiota analysis showed that the relative abundance of *Bifidobacterium* increased, whereas that of *Bacteroides* decreased. Correlation analysis suggested a significant association between increased *Bifidobacterium* level and reduced stress (*r* = −0.39, *p* = 0.0035) and insomnia levels (*r* = −0.3, *p* = 0.026).

**Conclusion:**

Prebiotics-containing granola improved subjective sleep quality and reduced stress in adults with stress-related sleep disturbances, which may be attributed to alterations in gut microbiota, particularly the increase in *Bifidobacterium* abundance.

## 1 Introduction

Sleep is essential for maintaining physiological homeostasis and cognitive functions. It regulates the autonomic nervous, cardiovascular, immune, and metabolic systems (1–4). Additionally, sleep plays a crucial role in cognitive performance, memory consolidation, and emotional regulation (5–7). However, sleep deprivation has become a widespread concern, particularly in Japan, where a significant proportion of the population reports insufficient sleep (8). Chronic sleep deprivation is associated with an increased risk of cardiovascular disease, diabetes, metabolic syndrome, and depression (9–11).

Among the various factors influencing sleep, stress is one of the most significant. Stress triggers physiological responses that increase alertness, potentially leading to primary or secondary sleep disorders (12–14). Acute and chronic stress-related insomnia affects a substantial proportion of the population, emphasizing the need for interventions that alleviate stress to improve sleep quality (15–18).

Recent evidence suggests a strong connection between the gut microbiota and brain function, known as the gut–brain axis (19). Stress alters gut microbiota composition, as demonstrated in animal models where restraint stress reduced *Bifidobacterium,* Ruminiclostridium and Lachnoclostridium and increased *Akkermansia* and *Faecalibaculum* levels (20). Conversely, gut microbiota may modulate stress responses. Germ-free mice, which lack gut microbiota, exhibit an exaggerated stress response, whereas colonization with normal gut microbiota restores stress resilience (21–23). Human studies also support this interaction; consumption of probiotic beverages has been shown to reduce perceived stress and lower salivary cortisol levels (24). One human study demonstrated that probiotic supplementation improved sleep quality and stress resilience. Medical students, who are generally assumed to be under chronic stress, were administered probiotic tablets containing *Bifidobacterium*. The intervention resulted in a reduction in subjective stress levels, improved Pittsburgh Sleep Quality Index (PSQI) scores, and decreased sleep latency (25). In addition, probiotic consumption has been shown to alter gut microbiota composition, reduce PSQI scores, increase total sleep time, and enhance mental well-being (26, 27). Additionally, prebiotic intake enhances short-chain fatty acid production and mitigates stress-induced hormonal responses (28). Prebiotics are non-digestible food components that selectively stimulate the growth and activity of beneficial gut bacteria, particularly *Bifidobacterium* and *Lactobacillus* (29). These compounds promote the production of short-chain fatty acids, such as butyrate, which have been linked to anti-inflammatory effects, stress regulation, sleep patterns and improved gut–brain communication (30, 31). Studies have shown that dietary interventions incorporating prebiotic-rich, indigestible food ingredients, formulated by a dietitian, lead to significant reductions in stress questionnaire scores and improvements in sleep quality (32). Given these insights, prebiotic intake may provide a novel approach to improving sleep and reducing stress-related insomnia. In this study, we formulated a granola containing multiple prebiotic compounds to evaluate their effects on gut microbiota composition, stress, and sleep quality. We aimed to determine whether prebiotic-containing granola consumption could alleviate stress and enhance sleep quality in individuals experiencing stress-related insomnia.

## 2 Materials and methods

### 2.1 Participants

Participants were recruited to this study through public calls in the form of posters, web pages, and emails from Clinical Creative (Sapporo, Japan). In total, 116 men and women aged 24–58 years participated in this study. We conducted a survey using the Epworth Sleep Scale, Athens Insomnia Scale, and Brief Job Stress Questionnaire. We prescreened 50 people using the following selection criteria: (1) those with an Epworth Sleepiness Scale score of ≥11 were excluded. (2) those judged to be under high stress based on the Brief Job Stress Questionnaire were included (score ≥ 77 in area B or total score ≥ 76 in areas A and C and ≥ 63 in area B). (3) If the total number of candidates who qualified after step (2) were > 50, the participants were selected in order of their score in area B. In case the scores were equal for area B, the participants were selected in the order of their scores on the Athens Insomnia Scale.

Next, the 50 pre-screened individuals were instructed to use an activity meter (Fitbit Inspire HR; Fitbit, San Francisco, CA, United States) to measure their sleep quality and sleep habits over a 7-day period. Additionally, they were required to complete the Profile of Mood States 2nd edition (POMS2), which is a questionnaire on defecation frequency (number of times of defecation/week), and a food frequency questionnaire (FFQ). We selected 27 volunteers (male, 12; female, 15) based on the following exclusion criteria: (1) had taken or had planned to take antibiotics, anti-allergy medications, sleeping pills, or sleeping aids in the month preceding the test date; (2) regular intake of supplements at a rate of more than three times/week (such as probiotic preparations and prebiotic supplements) that may affect the study outcome within the month preceding the test date; (3) history of serious diseases or current illnesses of the heart, liver, nervous system, digestive system, etc.; (4) incidence of chronic or acute serious infections; (5) scheduled to receive vaccinations during the study period; (6) pregnant or planning to become pregnant or breastfeeding; (7) habitual drinking of alcohol more than three times/week; (8) irregular eating habits; (9) body mass index (BMI) ≥ 30 (10) plans to significantly alter their lifestyle during the study period, such as traveling overseas to a different time zone (11); primary caretaker for persons requiring nursing care or an infant or may have their sleep disturbed by other external factors; (12) food allergies; (13) participation in a clinical study for other medicines or health foods or planning to participate in another clinical study less than 1 month after the end of this study or after providing consent to participate in the relevant study; (14) not engaged in full-time employment; (15) working in shifts or late night work; or irregular sleeping and waking times (with a difference of >5 h at bedtime); or extremely short or irregular sleep times (sleep for <4 h; as confirmed using Fitbit measurements); (16) non-possession of a smartphone; (17) unable to install the Fitbit applications on their smartphones; and (18) less than three bowel movements/week. Participants with diagnosed psychiatric disorders, such as schizophrenia, bipolar disorder, anxiety, or depression, were not explicitly excluded. However, we confirmed that none of the participants were taking medications commonly prescribed for these mental illnesses. Based on this criterion, we considered the study population to be free of psychiatric disorders. After the test, participants received an incentive. The sample size was determined based on the following. As it is known that the composition of the gut microbiota, a secondary evaluation item in this study, changes due to the intake of cereal (33), the effect size of this study was calculated using G*Power, assuming a large effect size (effect size 0.80). At a significance level of 0.05, the sample size required to achieve 95% power was *n* = 23. Furthermore, taking into account those who withdrew, were lost to follow-up, or were excluded from the analysis, we set the sample size at 27 participants per group. From the recruitment of participants to the completion of testing for all participants, this trial took place from November 2023 to June 2024. This study was conducted in accordance with the guidelines of the Declaration of Helsinki and was approved by the Ethics Committee of Sapporo Yuri no Kai Hospital (approval number: 029). All participants provided informed consent before enrolment in the study. This clinical trial was registered with the Institutional Ethics Committee of the Japanese University Hospital Medical Information Network (clinical trial reference number: UMIN 000053189).

### 2.2 Study design and procedure

**Figure 1:**
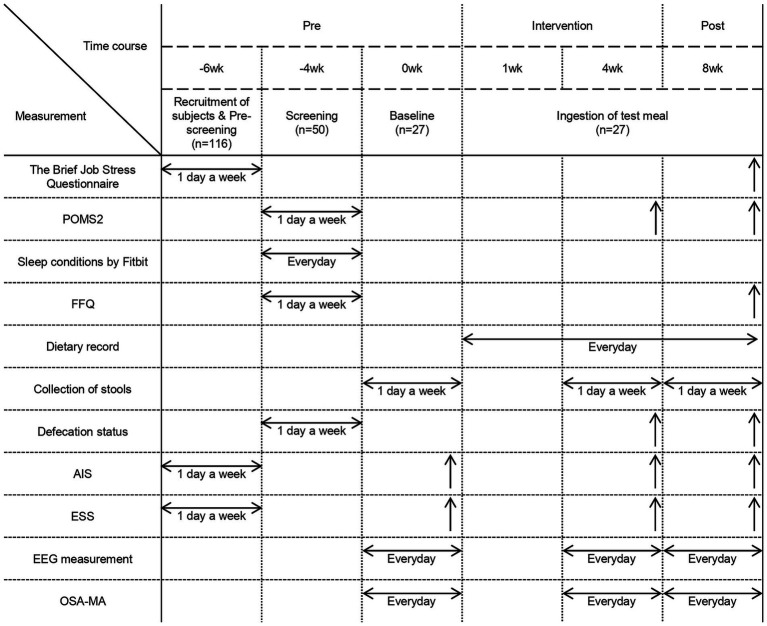
Experimental schedule. Experimental schedule and parameters measured during each period. POMS2: Profile of Mood States, 2nd Edition. FFQ, Food Frequency Questionnaire. AIS, Athens Insomnia Scale. ESS, Epworth Sleepiness Scale. EEG measurement, Electroencephalographic measurements. OSA-MA, Oguri-Shirakawa-Azumi Sleep Inventory, Middle-aged and Aged version. Measurements conducted over a period of time are represented by horizontal arrows, while those conducted on a specific day are represented by upward arrows, such as on the final day of the intervention.

The clinical trial was conducted in Hokkaido between April and June 2024 after the participants were selected for the single-arm uncontrolled single-blind before-and-after comparative study. Participants were informed that they would be consuming a cereal product as a test meal but were not aware of whether it contained prebiotics. This approach was implemented to minimize expectancy bias regarding the potential effects of prebiotics on sleep and stress. All participants were instructed to consume the test meals every day for 8 weeks, and record their meals including the consumption of the test meals in a food diary to ensure compliance with the intervention. Participants were allowed to consume granola once per day, and while breakfast was the recommended time, no specific instructions were given. All participants were required to complete a 7-day sleep measurement and answer the OSA-MA questionnaire at weeks 0 (1 week before the start of the study), 4 (4 weeks after the start of the study), and 8 (8 weeks after the start of the study). Additionally, the participants were instructed to use a Metabolokeeper® (Techno Suruga Labo Co., Ltd., Shizuoka, Japan) to collect their feces on 1 day of the following weeks: 0, 4, and 8. At the start of the study and on the last days of weeks 4 and 8, the participants were instructed to complete the questionnaires on defecation (number of times of defecation in a week and BSS for stool consistency), Athens Insomnia Scale, Epworth Sleep Scale, and POMS2. Additionally, the participants had to complete the Brief Job Stress Questionnaire and FFQ at the end of the study. Defecation frequency and stool consistency were recorded as an additional parameter to evaluate the effects of prebiotic intake on gut microbiota and gastrointestinal function. Since stress is known to affect gut function (34), we also examined whether changes in defecation frequency were associated with improvements in stress and sleep quality. All participants completed the questionnaires anonymously to ensure honest responses. The administration and data collection of the questionnaires were conducted by a third-party organization, ensuring impartiality. Before completing the questionnaires, participants received standardized instructions explaining the purpose of each item and how to respond appropriately. These measures were implemented to maintain the reliability and authenticity of the responses. The details of the study schedule are shown in Figure 1. The flow diagram of the participants in this study is shown in Supplementary Figure 1. There were no withdrawals among the participants who consumed the test meals, and all participants were used in the analysis. In this study, outcome measures were categorized into primary and secondary outcomes as follows. Primary Outcomes: The primary outcome measures included EEG-based sleep parameters, which served as objective indicators of sleep architecture and quality. These measures were selected as the primary indicators for evaluating the intervention’s impact on sleep. Secondary Outcomes: The secondary outcome measures included the OSA-MA questionnaire, the Athens Insomnia Scale, the Epworth Sleepiness Scale, POMS2, the Brief Job Stress Questionnaire, the Food Frequency Questionnaire (FFQ), questionnaires on defecation, and gut microbiota analysis. These measures were incorporated to assess subjective sleep quality and provide additional insights into various factors influencing sleep, such as insomnia severity, daytime sleepiness, mood states, occupational stress, dietary habits, and gut microbiota composition.

### 2.3 Test meals

The test meal comprised granola containing six types of prebiotic compounds (inulin, resistant starch, fructooligosaccharide, galactooligosaccharide, cacao mass, and barley). The consumption of each of these prebiotics alone increases short-chain fatty acid production (35–44). The granola contains approximately 1.4 g of each of the six prebiotics. The participants consumed 50 g of granola and 200 mL of milk daily. The brand of milk was specified to minimize the variation in the nutrient content of milk. The nutritional content of the test meal per serving (50 g) was 194.6 kcal; protein: 3.5 g; fat: 3.5 g; and carbohydrates: 41.2 g. This granola is already on the market, so the participants can eat it safely.

### 2.4 Sleep electroencephalography (InSomnograf®) measurement

S′UIMIN’s InSomnograf® is a device that measures the sleep stage based on EEG data obtained using five electrodes. These results show an 86.9% correlation with polysomnography (PSG) (45). Parameters related to sleep quality and quantity such as total sleep time (s), sleep onset latency (s), wakefulness after sleep onset (WASO; s), N1 total time (s), N2 total time (s), N3 total time (s), REM total time (s), and REM sleep rate (%) were calculated from the results of the sleep stage assessment (hypnogram) and used in the analysis. The participants wore the device immediately before bedtime to obtain EEG measurements. The average value was calculated for each of the 7 days of every week to determine the participant EEG parameter for the analysis.

### 2.5 16S rDNA gene sequencing and analysis

DNA was extracted from the collected feces using a revised version of a previously described method (46). The fecal sample (100 mg) was suspended in 4 M guanidine thiocyanate, 100 mM Tris–HCl (pH 9.0), and 40 mM EDTA, followed by bead-milling using a Precellys Evolution system (Bertin Instruments, FRA). DNA was extracted from the bead-treated suspension using the PI-480 and NR-201 systems (Kurabo Industries, Japan). The concentration and purity of the extracted DNA were measured using a spectrophotometer with the NanoDrop ND8000 instrument (Thermo Fisher Scientific, United States). The final DNA sample concentration was adjusted to 10 ng/μl.

The V3–V4 region of the 16S rDNA was amplified using the Pro341F/Pro805R primer set for prokaryotes. Primers were designed to complement the index primers provided by Illumina. The following primer sequences were used: Forward primer, 5’-AATGATACGGCGACCACCGAGATCTACACTCTTTCCCTACACGACGCTCTTCCGATCTCCTACGGGAGGCAGCAGCCTACGGGNBGCASCAG-3′; Reverse primer, 5’-CAAGCAGAAGACGGCATACGAGATNNNNNNGTGACTGGAGTTCAGACGTGTGCTCTTCCGATCTGACTACNVGGGTATCTAATCC-3′.

The touchdown PCR method for thermal cycling was used with a Rotor-Gene Q quantitative thermal cycler (Qiagen, Germany) to reduce the formation of spurious byproducts during the amplification process. The reaction mixture (25 μL) contained 10 ng of genomic DNA, MightyAmp for Real Time (SYBR Plus; Takara, Japan), and 0.25 μM of each primer. The PCR reaction conditions for DNA amplification were as follows: initial denaturation at 98°C for 2 min, followed by 35 cycles of annealing from 65°C to 55°C for 15 s, and extension at 68°C for 30 s. The annealing temperature was reduced by 1°C/cycle until the temperature was 55°C, which was maintained for the remaining cycles. The PCR products were purified using a MultiScreen PCRu96 filter plate (Merck Millipore, United States) and analyzed using a Bioanalyzer DNA 1000 Chip Kit (Agilent Technologies, United States) to detect primer dimers and determine the average molecular weight of each product. The purified products were quantified using real-time quantitative PCR (q-PCR) on a Rotor-Gene Q quantitative thermal cycler using MightyAmp for Real Time (SYBR Plus), 0.2 μM of each primer, which were derived from Illumina adapters, and serially diluted PhiX control library (Illumina, United States) as the standard. The PCR reaction conditions for quantification of each PCR product were as follows: initial denaturation at 98°C for 2 min, followed by 30 cycles of denaturation at 98°C for 10 s, annealing at 60°C for 15 s, and extension at 68°C for 30 s. A quantification step was performed to determine the concentration of the amplified libraries and confirm the presence of suitable primers for Illumina sequencing.

Sequencing was performed using MiSeq (Illumina, USA) and MiSeq Reagent Kit v3 (600 cycles). The sequenced 16S rDNA were processed using the quantitative Insights into microbiological Ecology 2 (QIIME2) pipeline (version 2024.2). The DADA2 algorithm was used to remove noise, perform quality filtering, and generate amplicon sequence variants (ASVs). The ASVs were assigned taxonomic groups from the phylum to genus levels using the Silva SSU Ref Nr 99 (version 132) classifier. Additionally, principal coordinate analysis (PCoA) was performed to determine alpha diversity, such as the Simpson diversity index, and beta diversity using weighted UniFrac distance.

### 2.6 Statistical analysis

#### 2.6.1 One-way repeated ANOVA or Friedman test

All statistical analyses were performed using GraphPad Prism version 9.0.2 (GraphPad Software Inc., San Diego, CA, United States). Data are expressed as mean ± SEM. The D’Agostino–Pearson test was used to assess the normality of the distributed data, whereas Bartlett’s test was used to examine whether the variation was equal or skewed. If the data showed a normal distribution and equal variation, statistical significance was determined using one-way repeated ANOVA with Tukey’s *post-hoc* test. If the data showed a non-normal distribution or biased variation, statistical significance was determined using Friedman’s and Dunn’s *post-hoc* tests. Statistical significance was set at *p* < 0.05.

#### 2.6.2 Correlation analysis

The D’Agostino–Pearson test was used to check for normal data distribution. Pearson’s correlation coefficient was calculated as a parametric test to check for correlation between normally distributed data. Spearman’s correlation coefficient was calculated as a non-parametric test to check for correlation between non-normally distributed data. We calculated the *p*-value for each test. A correlation coefficient of r < −0.2 or r > 0.2 was considered a significant correlation if *p* < 0.05.

#### 2.6.3 Multiple regression analysis

The value of each item was standardized using the STANDARIZE function in Excel, and analysis was performed using these standardized values. Additionally, the values for physical characteristics, such as age, BMI, sex, diastolic blood pressure, and systolic blood pressure, were calculation as adjustment variables. Furthermore, the VIF was calculation as a measure of multicollinearity, and it was confirmed to be ≤5.

## 3 Results

### 3.1 Survey of participant physical characteristics and food frequency questionnaire

**Figure 2:**
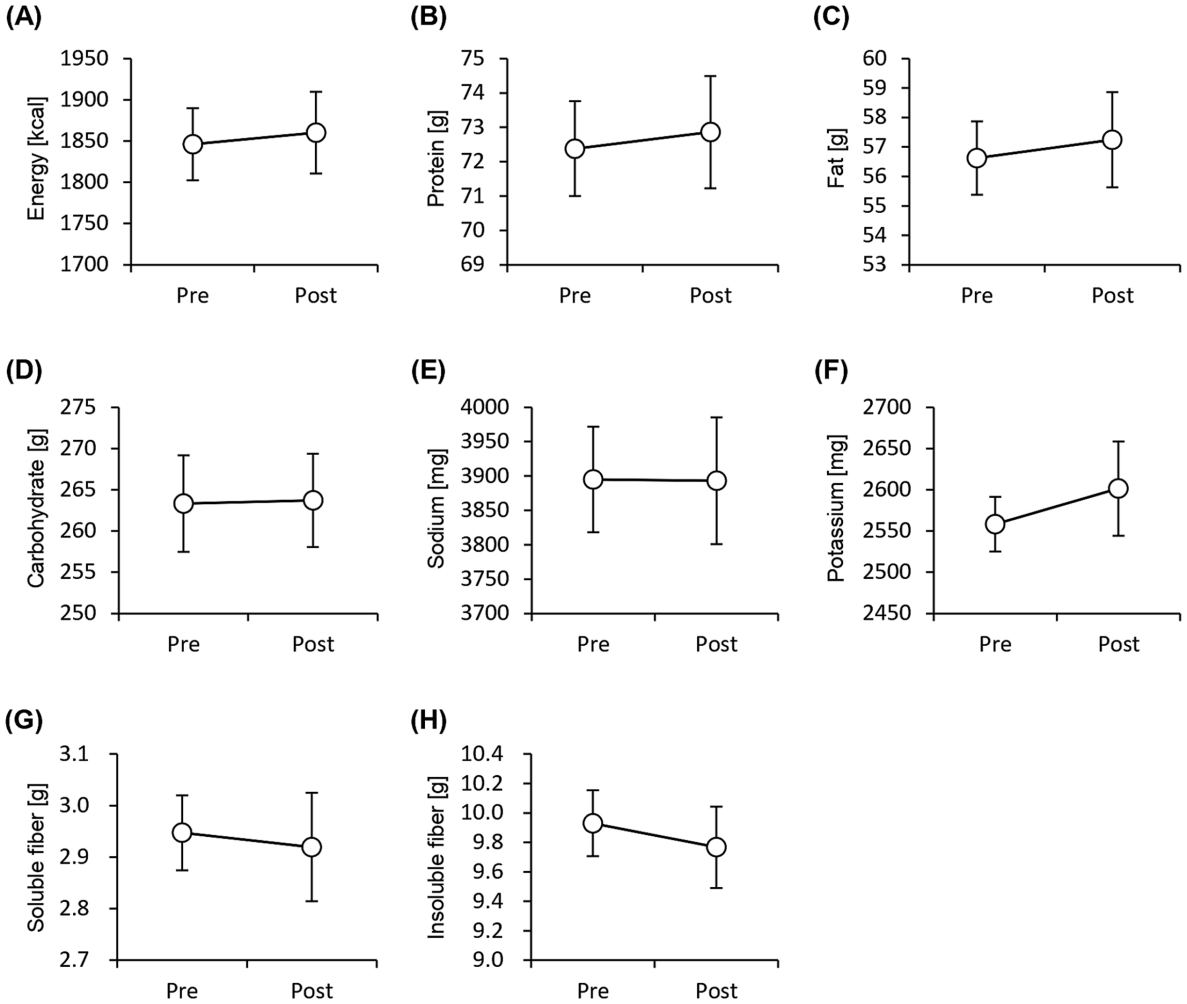
Frequency of nutrient intake does not change significantly before and after the study period. Daily consumption of nutrients was estimated using pre-period and post-period Food Frequency Questionnaires. **(A)** energy, **(B)** protein, **(C)** fat, **(D)** carbohydrate, **(E)** sodium, **(F)** potassium, **(G)** soluble dietary fiber, and **(H)** insoluble dietary fiber levels. All values are represented as mean ± SEM (*n* = 27; male *n* = 12, female *n* = 15).

The physical characteristics of the participants during the test period are listed in Table 1. The average age of the participants was 44.1 ± 1.50 years (mean ± SEM), and their average height was 167.1 ± 1.69 cm. The diastolic blood pressure reduced significantly after 8 weeks of consumption of granola containing several prebiotics. The quantity of nutrients consumed was calculated using the FFQ and compared between the pre- and post-tests; however, no significant difference was observed. This implies that the frequency of daily food intake did not change significantly during the test period (Figure 2).

**Table 1:** Physical characteristics of all participants.

	0 week	4 week	8 week	*p*-value
Weight (kg)	64.7 ± 2.74	64.7 ± 2.78	64.7 ± 2.71	0.9533
Body mass index	22.9 ± 0.630	22.9 ± 0.653	22.9 ± 0.619	0.9269
Systolic blood pressure (mmHg)	122.8 ± 1.98	119.0 ± 2.21	118.6 ± 2.47	0.0543
Diastolic blood pressure (mmHg)	79.5 ± 1.39	75.4 ± 1.47^**^	76.7 ± 1.73^*^	0.0079

All values are shown as mean ± SEM (*n* = 27; male *n* = 12, female *n* = 15). The *p*-value in the table indicate the *p*-value of the one-way repeated ANOVA test. **p < 0.01, *p < 0.05, evaluated using the one-way repeated ANOVA test with Tukey’s *post-hoc* test (vs. 0 week).

### 3.2 Consuming granola containing prebiotics improves participant sleepiness and insomnia

**Figure 3:**
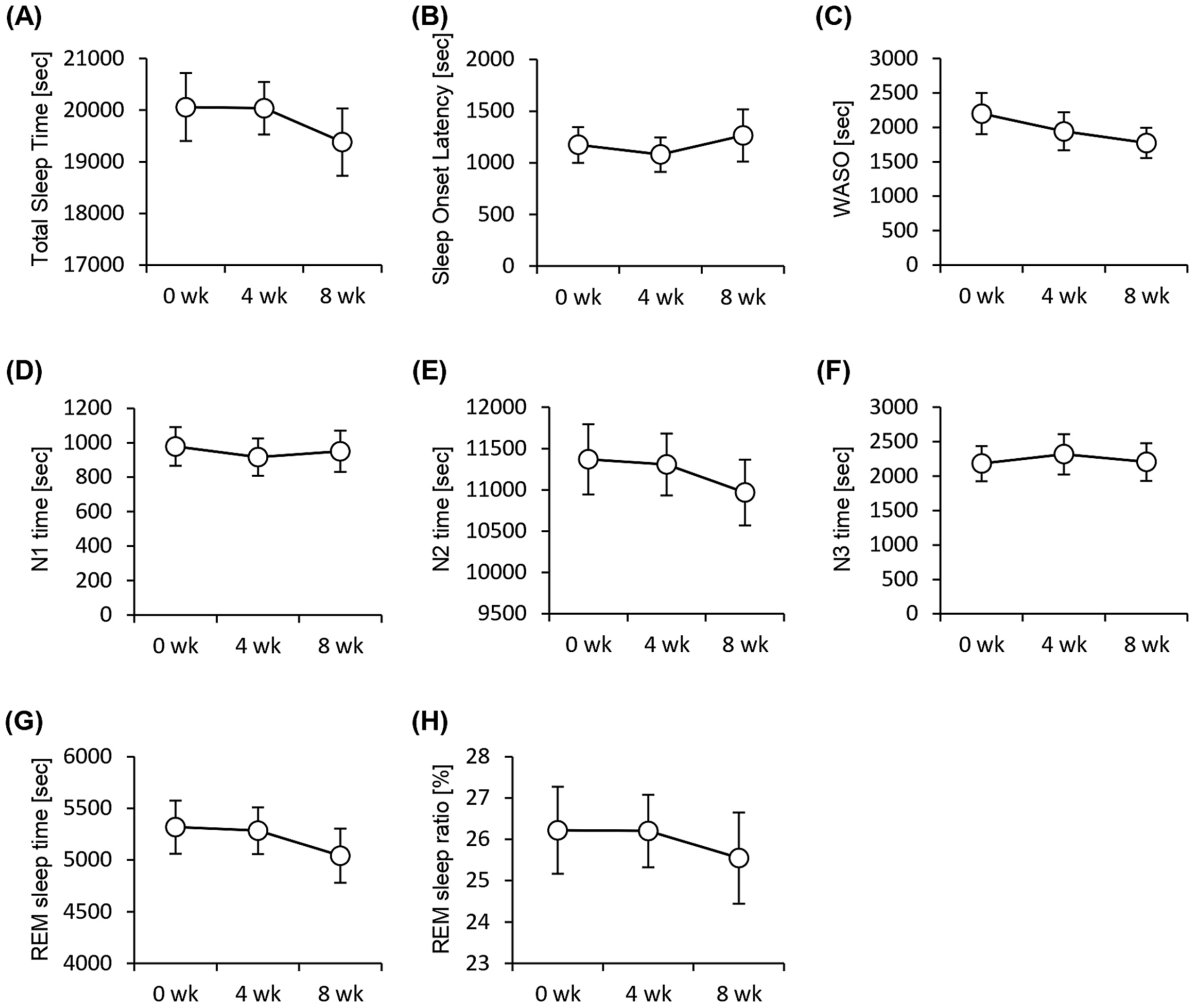
EEG measurement shows no significant difference in the sleep parameters. Summary of sleep parameters obtained from the EEG measurements. **(A)** Total Sleep Time. **(B)** Sleep onset latency, **(C)** WASO, wake time after sleep onset, **(D)** N1, non-REM sleep stage 1, **(E)** N2, non-REM sleep stage 2, **(F)** N3, non-REM sleep stage 3, **(G)** REM sleep time, and **(H)** REM sleep ratio. All values are represented as mean ± SEM (*n* = 27; male *n* = 12, female *n* = 15).

**Figure 4:**
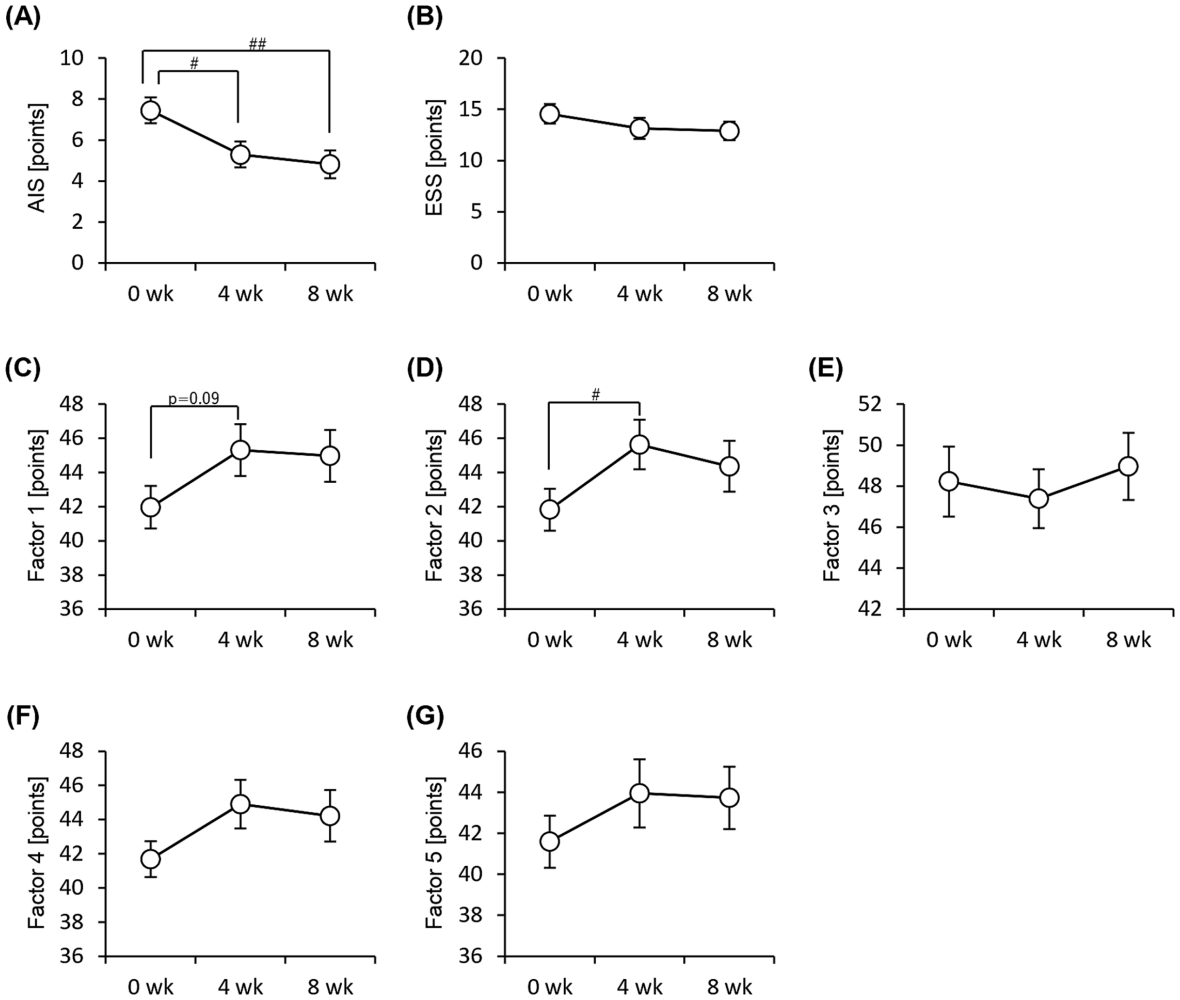
Subjective sleep quality improves with consumption of granola containing prebiotics. Summary of scores obtained from answers to each questionnaire. **(A)** AIS, Athens Insomnia Scale. **(B)** ESS, Epworth Sleepiness Scale. **(C–G)** OSA-MA, Oguri-Shirakawa-Azumi Sleep Inventory, Middle-age and Aged version [**(C)** Factor 1: sleepiness on rising, **(D)** Factor 2: initiation and maintenance of sleep, **(E)** Factor 3: frequent dreaming, **(F)** Factor 4: refreshing, **(G)** Factor 5: sleep length]. All values are represented as mean ± SEM (*n* = 27; male *n* = 12, female *n* = 15). ##*p* < 0.01, #*p* < 0.05, evaluated using the Friedman test with Dunn’s *post-hoc* test.

We performed an EEG to assess objective sleep status and administered the Athens Insomnia Scale, Epworth Sleepiness Scale, and OSA-MA questionnaires to assess subjective sleep status. None of the sleep-state parameters obtained from EEG measurements showed significant differences (Figure 3). In contrast, the Athens Insomnia Scale for the subjective sleep parameters showed significant lower scores at both weeks 4 and 8 after the consumption of prebiotic granola compared with that at week 0 (Figure 4). Additionally, the score for Factor 1 (sleepiness on rising) on the OSA-MA showed an increasing trend on week 4, and the score for Factor 2 (initiation and maintenance of sleep) increased significantly (Figures 4C,D). These results suggest that the consumption of prebiotics-containing granola improves subjective sleepiness and insomnia. Here, we examined the correlation between an objective sleep indicator, electroencephalogram (EEG) measurements, and subjective sleep questionnaire evaluations. The correlation analysis showed a positive correlation between subjective sleep time and objective sleep time [Factor 5-TST (*r* = 0.27)] and a negative correlation between subjective sleep onset and sleep maintenance and objective middle of the night awakening and sleep latency [Factor 2-WASO (*r* = −0. 2), Factor 2-SOL (*r* = −0.27)]. Although subjective and objective ratings showed a correlation, subjective sleepiness (AIS) and objective indicators did not show any correlation, and inconsistent results were obtained (Supplementary Figure 2).

### 3.3 Consumption of prebiotics-containing granola improves stress and total mood disturbance

**Figure 5:**
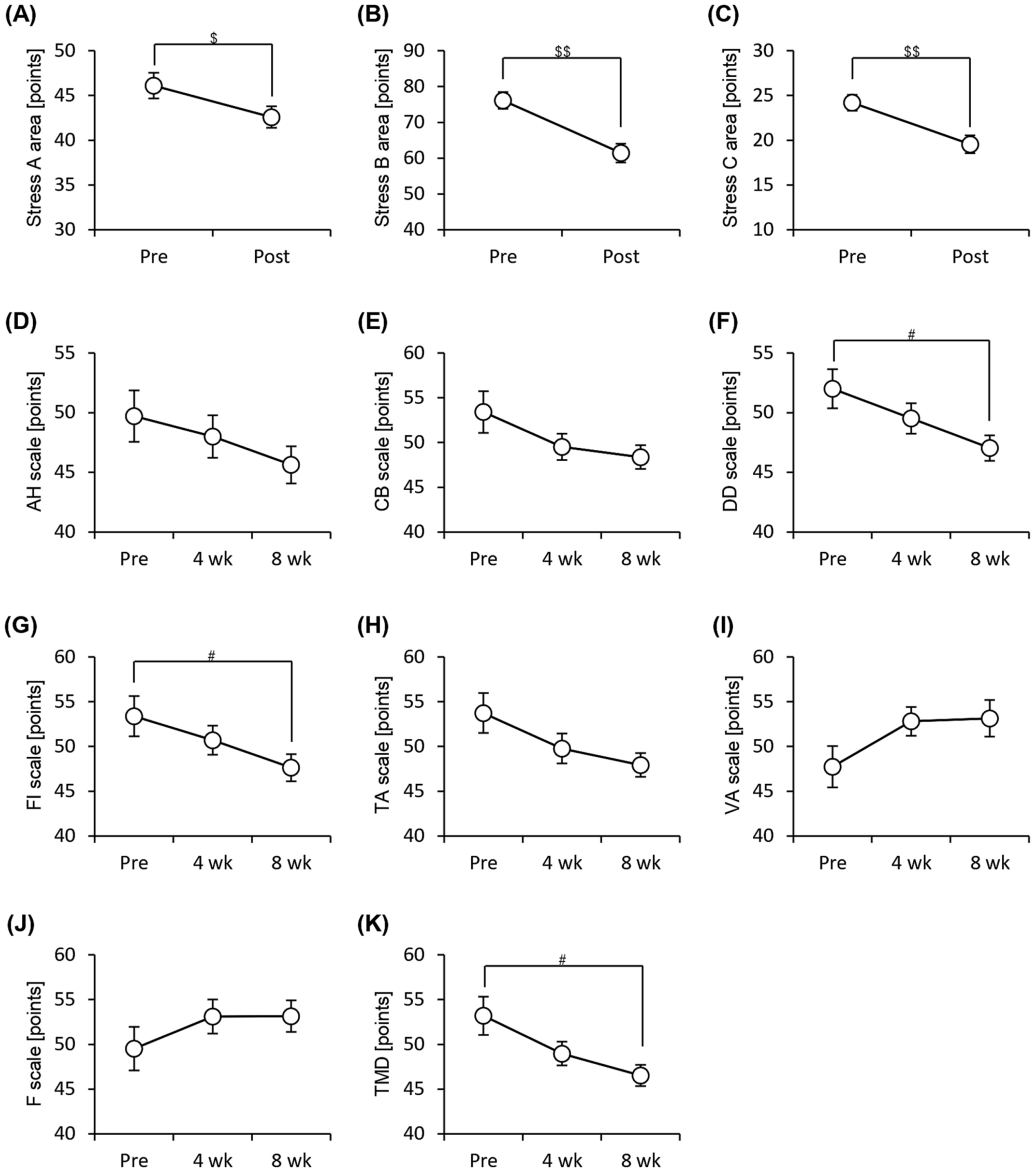
Subjective stress and mood states improve with consumption of granola containing prebiotics. Summary of scores obtained from answers to each questionnaire. **(A–C)** Brief Job Stress Questionnaire [**(A)** Stress area A, job stressors, **(B)** Stress area B, psychological and physical stress reactions, and **(C)** Stress area C, buffering factors such as social support at work], **(D–K)** POMS2, Profile of Mood States 2nd edition [**(D)** AH, anger–hostility scale; **(E)** CB, confusion–bewilderment scale; **(F)** DD, depression–dejection scale; **(G)** FI, fatigue–inertia scale; **(H)** TA, tension–anxiety scale; **(I)** VA, vigor–activity scale, **(J)** F, friendliness scale, **(K)** TMD, total mood disturbance]. All values are represented as mean ± SEM (*n* = 27; male *n* = 12, female *n* = 15). ##*p* < 0.01, #*p* < 0.05, evaluated using the Friedman test with Dunn’s *post-hoc* test. $$p < 0.01, $p < 0.05, evaluated using Wilcoxon signed-rank test.

We administered the Brief Job Stress Questionnaire and the POMS2 to examine the stress and psychological mood states of the participants. The scores for all three areas (area A: job stressors, area B: psychological and physical stress reactions, and area C: social support at work) were significantly low after 8 weeks of consuming the prebiotics-containing granola (Figures 5A–C). The POMS2 scores on the DD (depression–dejection) and FI (fatigue–inertia) scales decreased significantly after 8 weeks of consuming prebiotics-containing granola. Additionally, the total mood disturbance (TMD) score calculated based on the six POMS2 scales decreased significantly (Figures 5D–K). These results suggest that the consumption of prebiotics-containing granola improves stress and reduces feelings of depression, fatigue, and lethargy, thereby reducing psychological mood disturbances. Additionally, we examined the number of bowel movements and stool characteristics; however, no significant differences were observed (Supplementary Figure 3).

### 3.4 Consumption of prebiotics-containing granola increases the relative abundance of *Bifidobacterium* and decreases that of *Bacteroides*

**Figure 6:**
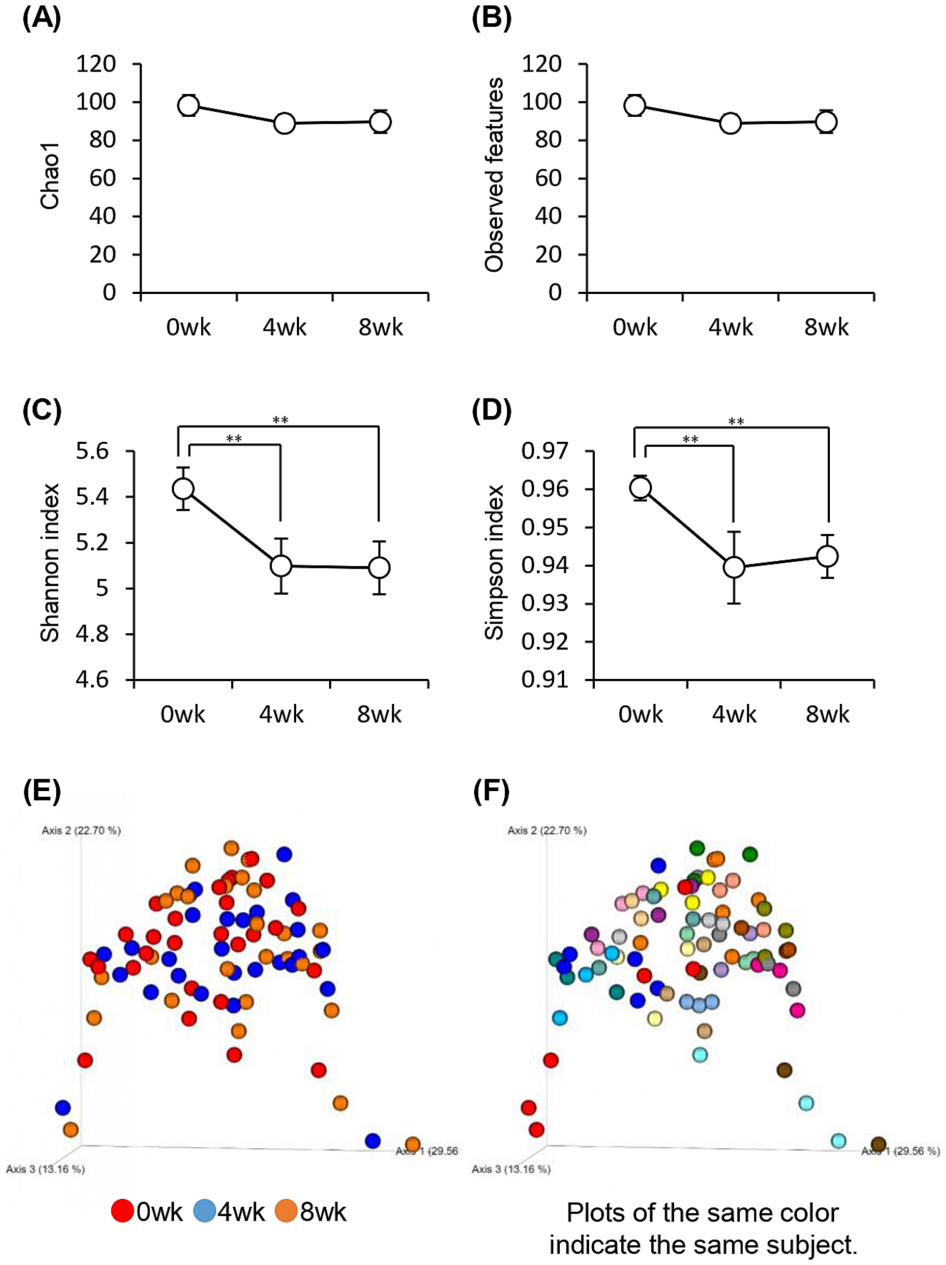
Consuming prebiotics-containing granola reduced intestinal microbiota diversity. **(A–D)** Bacterial alpha diversity; comparison of **(A)** Chao1, **(B)** Observed features, and **(C)** Shannon index. **(D)** Simpson index-based estimation of the 16S rDNA gene libraries at 99% similarity to sequencing analysis. **(E–F)** Bacterial beta diversity comparison by **(E)** week or **(F)** individual. All values are represented as mean ± SEM (*n* = 27; male *n* = 12, female *n* = 15). **p < 0.01, evaluated using the one-way repeated ANOVA with Tukey’s *post-hoc* test.

We investigated the changes in the gut microbiota associated with the consumption of prebiotics-containing granola. The alpha diversity of the gut microbiota reduced significantly, as indicated by the Shannon and Simpson diversity indices (Figures 6A–D). However, the Chao1 diversity index and Observed_features, which strongly reflects the number of bacterial species, did not differ significantly. Thus, the results suggest that the decline in gut microbiota diversity may be attributed to a decrease in the evenness of the microbiota rather than a decrease in the number of species.

The composition of the gut microbiota was measured using beta diversity. The results were compared weekly; however, no significant difference was observed between each week (*p* = 0.74), and the composition of the gut microbiota did not change significantly even when the prebiotics-containing granola was consumed. However, comparison of the gut microbiota between individuals showed significant differences (*p* = 0.001), implying that the difference in gut microbiota composition was more because of variation between individuals than because of the consumption of prebiotics-containing granola (Figures 6E,F).

**Figure 7:**
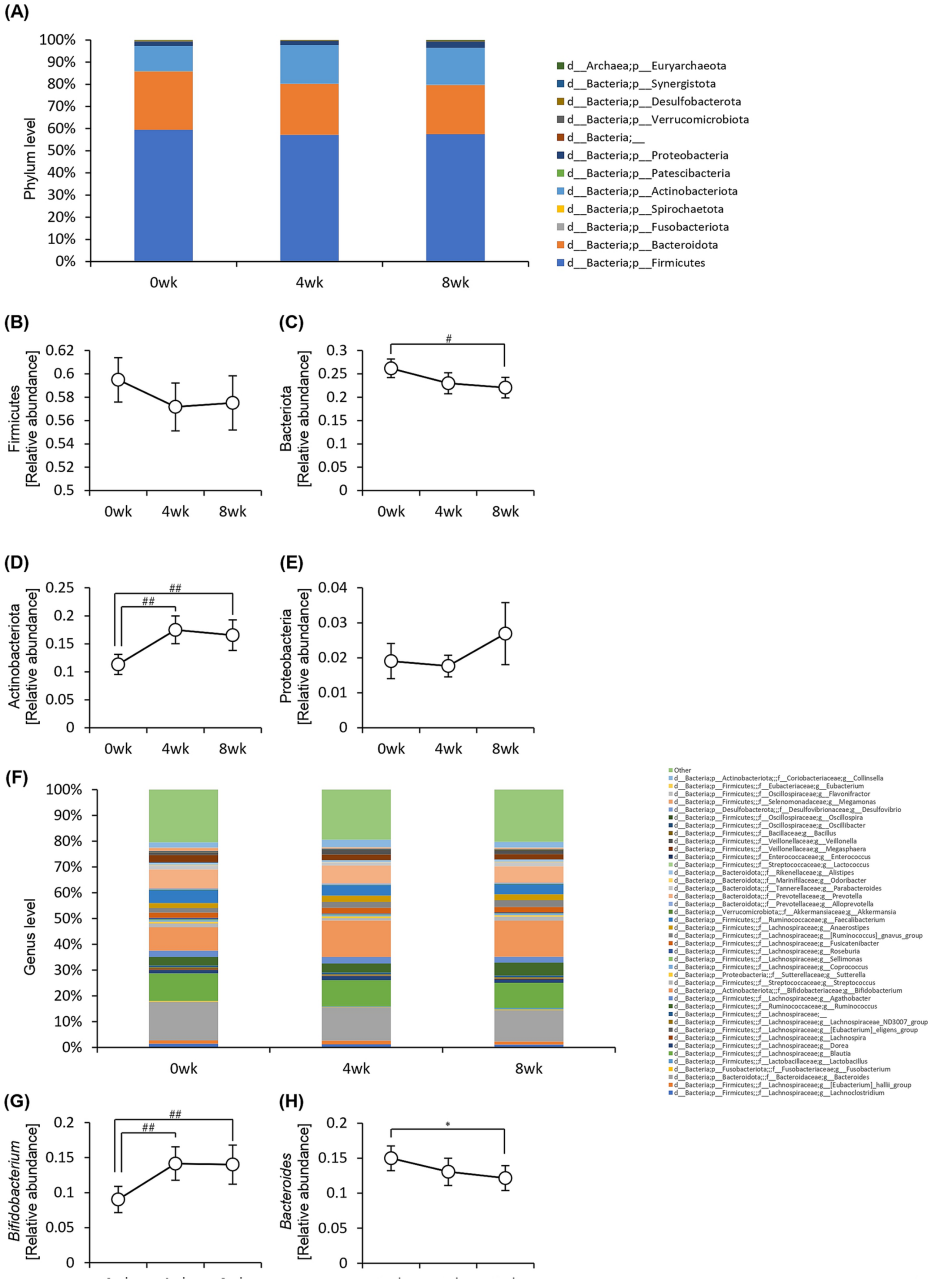
Consumption of prebiotics-containing granola increased the relative abundance of *Bifidobacterium* and reduced that of *Bacteroides*. **(A)** Composition of intestinal microbiota at phylum level. **(B–E)** Relative abundance of microbes at phylum level [**(B)** Firmicutes, **(C)** Bacteriota, **(D)** Actinobacteriota, **(E)** Proteobacteria]. **(F)** Composition of the intestinal microbiota at genus level. (G–H) Relative abundance of microbes at genus level [**(G)** *Bifidobacterium*, **(H)** *Bacteroides*]. All values are represented as mean ± SEM (*n* = 27; male *n* = 12, female *n* = 15). *p < 0.05, evaluated using the one-way repeated ANOVA with Tukey’s *post-hoc* test. ##*p* < 0.01, #*p* < 0.05, evaluated using the Friedman test with Dunn’s *post-hoc* test.

We examined the relative abundance of gut bacteria at the phylum (Figure 7A) and genus levels (Figure 7F). At the phylum level, the relative abundance of Bacteroidota significantly decreased, whereas that of Actinobacteriota significantly increased after 8 weeks of consumption of prebiotics-containing granola (Figures 7B–E). At the genus level, the relative abundance of *Bifidobacterium* increased, whereas that of *Bacteroides* significantly decreased (Figures 7G,H).

### 3.5 Increasing the relative abundance of *Bifidobacterium* may improve mental and physical stress response

**Figure 8:**
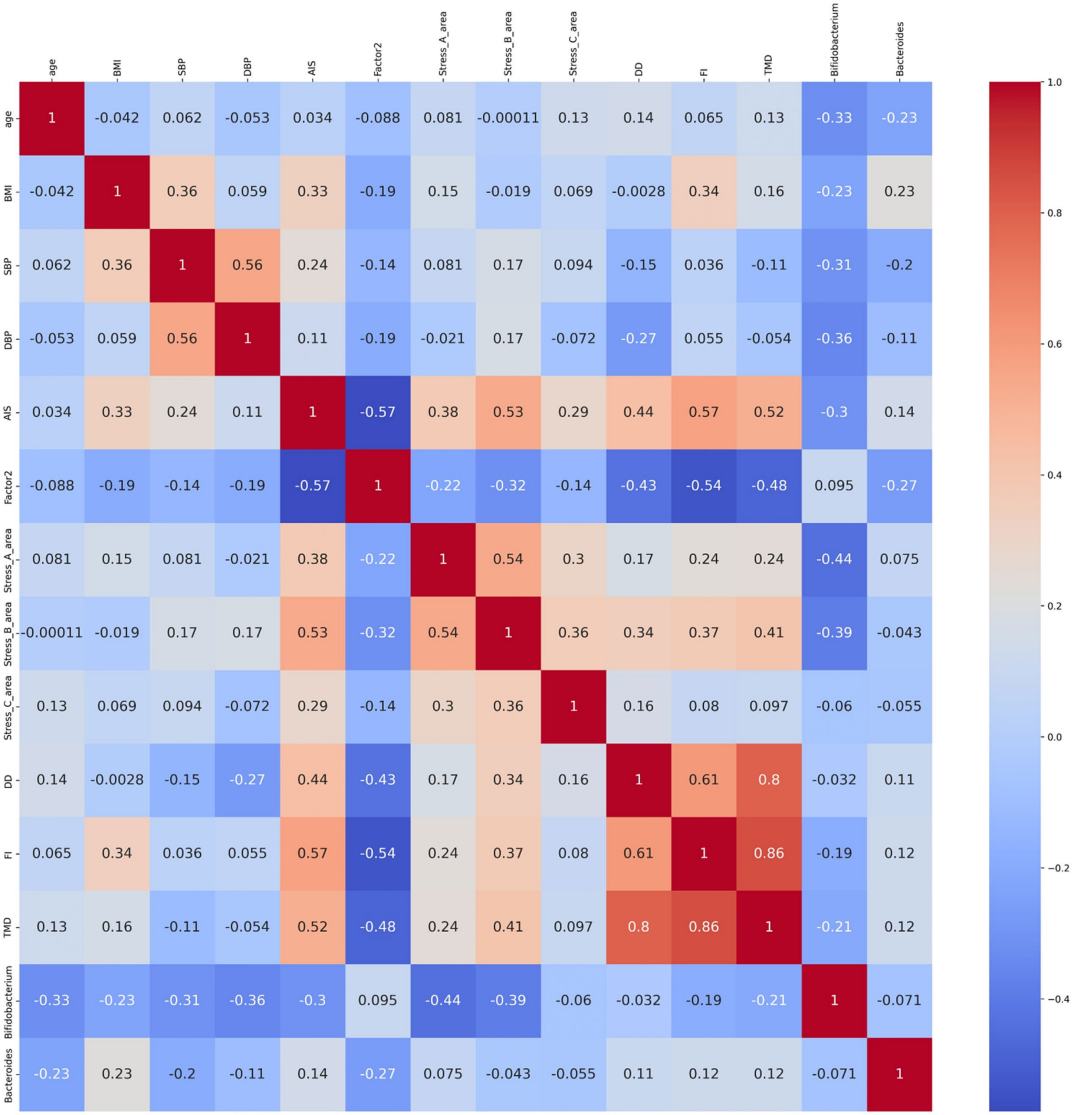
*Bifidobacterium* showed correlation with sleep and mood states. Heatmap of the correlation between age; BMI, body mass index; SBP, systolic blood pressure; DBP, diastolic blood pressure; AIS, Athens Insomnia Scale; ESS, Epworth Sleepiness Scale; OSA-MA (Factor 2; initiation and maintenance of sleep); Brief Job Stress Questionnaire (stress area A: job stressors, stress area B: psychological and physical stress reactions, and stress area C: buffering factors such as social support at work); and POMS2 (DD: depression–dejection scale, FI: fatigue–inertia scale; TMD: total mood disturbance), *Bifidobacterium*, and *Bacteroides*. The numbers in the diagram represent the correlation coefficients, which were calculated using the Spearman’s correlation coefficient (*n* = 27; male *n* = 12, female *n* = 15).

We conducted a correlation analysis to explore the relationship between the gut microbiota and subjective sleep indicators, as measured by various questionnaires (Figure 8). This analysis included the Athens Insomnia Scale; Factors 2 of the OSA-MA; Areas A, B, and C of the Brief Job Stress Questionnaire; DD scale; FI scale; TMD, *Bifidobacterium*, and *Bacteroides* of the POMS2. A correlation matrix was created. The values obtained from the pre-period and week 8 (post) were used in the correlation analysis, whereas the values obtained from week 0 in the pre-period were used for AIS and ESS. Age, BMI, diastolic blood pressure, and systolic blood pressure were also included as physical characteristics in the correlation matrix. We focused on relationships where the absolute value of the correlation coefficient was >0.2. *Bifidobacterium* showed a significant negative correlation with AIS, areas A and B, and TMD. Additionally, we detected a significant negative correlation between *Bacteroides* and Factor 2 (Figure 8). As positive correlation was observed between AIS and BMI and negative correlation between *Bifidobacterium* and systolic blood pressure, the influence of physical characteristics was considered, and multiple regression analysis was performed to investigate in detail the relationship between gut bacteria and subjective sleep indicators. For the multiple regression analysis, AIS, Areas A and B, and TMD were used as objective variables, *Bifidobacterium* as the explanatory variable, and physical characteristics as the adjustment factor. *Bifidobacterium* showed a negative association with AIS and TMD, and a significant negative association with areas A and B (Table 2). However, no significant association was observed between *Bacteroides* and Factor 2 (Table 3). These results suggest that the consumption of prebiotics-containing granola may increase the relative abundance of *Bifidobacterium* and contribute to reductions in mental and physical stress. While not statistically significant, this increase also showed a tendency to improve subjective insomnia and mood states.

**Table 2:** Multiple regression analysis using *Bifidobacterium* as explanatory variable.

	Objective value			
	AIS	A_area	B_area	TMD
R^2^-value	0.0943	0.0669	0.0527	0.0169
*Bifidobacterium*	−0.270 [−0.584–0.045] (0.061)	−0.418* [−0.737–−0.099] (0.011)	−0.399* [−0.720–−0.077] (0.0162)	−0.285 [−0.612–0.043] (0.066)
Age	0.0016 [−0.310–0.313] (0.568)	−0.011 [−0.326–0.305] (0.946)	−0.181 [−0.499–0.138] (0.260)	0.215 [−0.225–0.423] (0.541)
Sex_id	−0.252 [−0.995–0.492] (0.499)	−0.306 [−1.061–0.449] (0.419)	0.252 [−0.508–1.013] (0.507)	−0.386 [−1.161–0.389] (0.321)
BMI	0.095 [−0.250–0.440] (0.582)	−0.018 [−0.368–0.331] (0.916)	−0.149 [−0.502–0.203] (0.399)	0.073 [−0.286–0.432] (0.684)
SBP	0.226 [−0.195–0.650] (0.286)	0.114 [−0.314–0.542] (0.595)	0.228 [−0.203–0.660] (0.292)	−0.214 [−0.653–0.225] (0.332)
DBP	−0.084 [−0.471–0.304] (0.665)	−0.189 [−0.582–0.204] (0.339)	−0.085 [−0.481–0.312] (0.669)	−0.0061 [−0.410–0.398] (0.976)

The numbers are partial regression coefficients, the numbers in brackets are 95% confidence intervals, and the numbers in parentheses are *p*-values, **p < 0.01, *p < 0.05 (*n* = 27; male *n* = 12, female *n* = 15). AIS, Athens Insomnia Scale. A_area, Area A in the Brief Job Stress Questionnaire (job stressors). B_area, Area B in the Brief Job Stress Questionnaire (psychological and physical stress reactions). TMD, Total mood disturbance. BMI, Body mass index. SBP, Systolic blood pressure. DBP, Diastolic blood pressure.

**Table 3:** Multiple regression analysis using *Bacteroides* as explanatory variable.

	Objective value
	Factor 2
R^2^-value	0.0106
*Bacteroides*	−0.209 [−0.547–0.130] (0.221)
Age	−0.192 [−0.526–0.141] (0.251)
Sex_id	−0.020 [−0.880–0.841] (0.964)
BMI	−0.069 [−0.470–0.332] (0.730)
SBP	−0.082 [−0.522–0.358] (0.710)
DBP	−0.146 [−0.527–0.236] (0.447)

The numbers are partial regression coefficients, the numbers in brackets are 95% confidence intervals, and the numbers in parentheses are *p*-values, **p < 0.01, *p < 0.05 (*n* = 27; male *n* = 12, female *n* = 15). Factor 2: Factor 2 on the OSA-MA (initiation and maintenance of sleep). BMI, Body mass index. SBP, Systolic blood pressure. DBP, Diastolic blood pressure.

## 4 Discussion

In this study, although the subjective sleep index improved, none of the objective EEG measures showed significant changes. We examined the correlation between subjective and objective sleep indicators. While a strong relationship was observed in some cases, no correlation was found in others, resulting in inconsistent findings. Poor subjective sleep quality is often closely associated with short total sleep time and frequent middle-of-the-night awakening (measured objectively) (47–50). A study compared the Athens Insomnia Scale scores of healthy adult men and women who were sorted into good, moderate, and poor sleep quality groups. The sleep quality was identified using cluster analysis of the proportion of each sleep stage determined based on sleep EEG, sleep latency, midnight awakenings, total sleep time, and subjective insomnia disturbance. No significant differences were observed between the groups (51). Systolic blood pressure was examined and found to be significantly high in the poor sleep group. Although the objective sleep indicators showed a correlation with systolic blood pressure, no correlation was observed with the Athens Insomnia Scale, which is a subjective sleep indicator (51). Moreover, comparing the depression symptom scores using the Athens Insomnia Scale between patients with major depression, schizophrenia, bipolar disorder, and anxiety disorder who were sorted into insomnia and non-insomnia groups showed that the scores were significantly high in the insomnia group; however, when divided into insomnia and non-insomnia groups based on sleep EEG, no differences were observed in the depression symptom scores (52). These studies suggest that objective and subjective sleep indicators may quite possibly diverge without correlation. Furthermore, the items of objective and subjective sleep indicators show high variability; hence, no consistent objective indicator is available for judging subjective sleep quality (53). Therefore, subjective measures depend on an individual’s cognitive and psychological states, whereas EEG reflects physiological processes. These differences in cognitive and physiological responses are thought to exert an effect. Of course, since this study employed a single-group pre-post comparison design, the possibility of a placebo effect or psychological expectancy cannot be ruled out. Participants’ awareness of the intervention may have influenced their perception, leading them to subjectively report improved sleep quality. In addition, an intervention study using electronic noise-masking earbuds for medical professionals with sleep disorders reported a greater improvement in subjective questionnaire responses than in objective EEG measures (54). In other words, objective sleep evaluation may be more sensitive to intervention effects than subjective sleep evaluation. To assess the sensitivity of the EEG measurements, a *post-hoc* power analysis was conducted using G*Power. The analysis revealed that, given our sample size (*N* = 27) and the assumed effect size (*d* = 0.3), the achieved statistical power was 0.92. This indicates that our EEG analysis had sufficient sensitivity to detect potential changes in sleep parameters. Therefore, the absence of significant changes in EEG metrics suggests that the intervention may not have induced measurable alterations in objective sleep architecture, rather than the results being limited by insufficient statistical power.

In this study, the consumption of prebiotics-containing granola improved stress response and total mood disturbance. As mentioned previously, both subjective and objective stress indicators exist. The two major methods for objectively assessing stress are biochemical methods that analyze the components of blood, urine, and saliva and physiological methods that analyze biological signals such as heart rate variability and respiratory activity (55). However, cortisol in the blood and urine, which is used as a stress marker, responds to acute stress (55) and would probably be unsuitable for evaluating stress fluctuations over an 8-week period, as was set in this study. Moreover, salivary stress markers, including cortisol, are affected by circadian rhythms; therefore, collecting samples at regular intervals is vital (56). Previous studies have reported a positive correlation between heart rate variability and subjective stress indicators, even after adjusting for confounding factors such as sex, age, physical activity, and body fat percentage. However, subjective stress and physical activity were correlated, and a strong relationship was reported between heart rate variability and age; therefore, heart rate variability may be influenced by other factors (57). Although subjective and objective indicators of stress do not always correlate, measuring several objective indicators may allow us to further investigate the relationship between stress, gut microbiota, and sleep. Previous studies have reported mixed findings on the relationship between objective and subjective stress indicators. For example, while some studies have identified a positive correlation between heart rate variability and perceived stress levels, others have found no significant association after adjusting for confounding factors such as sex, age, physical activity, and autonomic nervous system function (57, 58). One possible explanation for these inconsistencies is that objective stress indicators, such as heart rate variability and cortisol levels, are influenced by multiple physiological and environmental factors. Heart rate variability is highly sensitive to acute stressors but may not always reflect chronic stress fluctuations over extended periods. Similarly, cortisol levels exhibit circadian variation and can be affected by factors such as sleep patterns, physical activity, and dietary intake (55, 56). Additionally, individual differences in autonomic nervous system regulation and stress resilience may contribute to variations in physiological stress responses (59). In this study, we did not measure objective stress indicators such as heart rate variability or cortisol levels; therefore, we cannot directly assess their relationship with subjective stress measures. Future research incorporating both subjective and objective stress indicators will be essential to further clarify their interrelationship and the potential impact of prebiotic interventions on physiological stress responses.

In this study, when prebiotic-containing granola was consumed for 8 weeks, diastolic blood pressure began to decrease from the fourth week. Previous studies have reported that granola consumption significantly reduces both diastolic and systolic blood pressure in hemodialysis patients (60). In particular, *β*-glucan, a component of granola, has been shown to improve cardiovascular risk factors, including blood pressure (61). One proposed mechanism by which dietary fiber intake lowers blood pressure involves short-chain fatty acids (SCFAs). SCFAs produced by gut bacteria are thought to activate GPR41, which in turn lowers blood pressure by reducing sympathetic nerve activity (62). Alternatively, SCFAs may act on vascular endothelial cells, activating endothelial nitric oxide synthase (eNOS) to produce nitric oxide, which induces vasodilation and lowers blood pressure (63, 64). Although these pathways are believed to play a role, the precise reason why only diastolic blood pressure decreased significantly in this study remains unclear. One possibility is that different types of stress influence diastolic and systolic blood pressure differently (65, 66). For example, one study reported that under calculation task-induced stress, depressive symptoms were associated with changes in systolic but not diastolic blood pressure (67). This suggests that the responses of diastolic and systolic blood pressure vary depending on stress conditions. In this study, many participants exhibited a tendency toward higher diastolic blood pressure, which may explain why significant reductions in diastolic blood pressure were observed following granola consumption.

In this study, consuming prebiotics-containing granola for 8 weeks significantly reduced some indicators of alpha diversity in the gut microbiota, resulting in lower gut microbiota diversity. Despite this reduction in alpha diversity, subjective feelings of insomnia and mental and physical stress responses improved. The use of antibiotics and unbalanced diet reduce the diversity of the gut microbiota, and a reduction in gut microbiota diversity is associated with several diseases, including obesity, type 2 diabetes, nonalcoholic liver disease, and heart disease (68–70). Additionally, reduced alpha diversity has been reported in patients with depression and insomnia (71–73). However, some studies suggest that alpha diversity is higher in people with depression (74), whereas others suggest no link between alpha diversity and depression (75). The correlation among the Pittsburgh Sleep Quality Index, sleep apnea score, and alpha diversity was analyzed in patients with chronic insomnia, but no significant correlation was detected (76). This implies that no consistent association exists between gut microbiota diversity and disorders such as sleep and stress responses, and perhaps decrease in alpha diversity is not associated with improvements in insomnia or stress responses. Although some studies have reported that feeding prebiotics and probiotics increased the Alpha Diversity Index, others have reported that it decreased it; thus, the results are inconsistent (77–80). Various factors such as the sex and presence of absence of diseases may have contributed to this inconsistency. In the current study, the composition of the gut microbiota may have shown a trend toward a certain convergence direction as a result of the consumption of prebiotics-containing granola, which is not normally eaten for breakfast for 8 weeks; furthermore, and the diversity index may have decreased as a result. However, consuming probiotics has been shown to maintain alpha diversity in response to stress (24); therefore, further studies are warranted to clarify the relationship between changes in gut microbiota diversity due to prebiotic consumption and stress responses and sleep.

In this study, the relative abundance of *Bifidobacterium* increased after 8 weeks of consuming prebiotics-containing granola, and a significant association was observed between *Bifidobacterium* and stress responses and subjective insomnia. In this study, although many elements of Area A in the Brief Job Stress Questionnaire cannot be directly modified, stress perception and experience may still vary within a fixed occupational environment, potentially leading to a decrease in Area A scores. In humans, emotional stress causes short- and long-term decrease in *Bifidobacterium* abundance (81). Patients with bipolar disorder showed significant negative correlation between *Bifidobacterium* counts and cortisol levels, which implies that *Bifidobacterium* may be involved in stress response (82). Among Japanese individuals, middle-aged and older women with functional constipation reported longer WASO times and lower *Bifidobacterium* population (83). Moreover, *Lactobacillus gasseri* CP2305 administration to Japanese medical students taking the National Medical Practitioners Qualifying Examination significantly reduced anxiety and sleep disturbances and suppressed stress-induced *Bifidobacterium* reduction (25). *Bifidobacterium adolescentis* SBT2786 administration increased the duration of sleep time in healthy Japanese men and women between the ages of 30 and 59 who were dissatisfied with their quality of sleep; however, it increased the duration of light sleep and did not improve their subjective sleep quality. In contrast, it improved their mood. Additionally, a subgroup analysis of participants with high stress levels showed increase in sleep time and improvement in sleepiness on rising (84). *Bifidobacterium breve* M-16 V administration reduced heart rate under stress and improved mood and sleep scores in participants with high anxiety levels (85). Thus, increasing *Bifidobacterium* abundance may be beneficial for mental and physical stress symptoms, sleep quality, and mood. Several studies have focused on prebiotics rather than probiotics, suggesting that galacto-oligosaccharides and inulin may reduce stress responses (86, 87) and that yeast mannan may increase N3 sleep duration (88). However, none of these studies have reported an increase in *Bifidobacterium* following prebiotic consumption. Additionally, most studies examining the relationship between sleep and gut microbiota have focused on probiotics, with relatively few investigating prebiotics (89). Therefore, future research is needed to determine whether prebiotic consumption increases *Bifidobacterium* and influences sleep and stress.

Several studies have reported that probiotic supplementation reduces subjective stress, improves subjective sleep quality, and reduces anxiety and depression-like behavior (90–92). The possible pathways through which gut bacteria regulate stress and sleep include changes in gut hormones, gut-associated peptides, and the vagus nervous system (93, 94). According to this study, prebiotics-mediated regulation of these pathways may be specifically related to the production of short-chain fatty acids by the gut microbiota. Short-chain fatty acids such as butyric acid and propionic acid affect the afferent vagus nerve system, which transmits information to the brain (95–97). Stimuli transmitted to the brain via the vagus nerve are relayed to the limbic system and hypothalamus through the solitary nucleus (98, 99). This process may help alleviate stress responses by influencing the secretion of serotonin and dopamine (100). This reduces stress responses and affects sleep quality. Furthermore, short-chain fatty acids stimulate the secretion of the gastrointestinal hormones GLP-1 and PYY (101, 102). The secreted GLP-1 is believed to cross the blood–brain barrier and act on GLP-1 receptors, with the stimulation of these receptors being processed through the amygdala and influencing emotional behavior (103). On the other hand, PYY is believed to influence the brain through indirect signaling via the vagus nerve rather than by crossing the blood–brain barrier (104). As *Bifidobacterium* promotes the production of acetic and butyric acids (105, 106), an increase in its relative abundance may increase the quantity of short-chain fatty acids in the intestinal tract. Additionally, *Bifidobacterium adolescentis* possesses a particularly high gamma-aminobutyric acid (GABA) producing capacity among the *Bifidobacterium* genus (107). GABA produced in the gut affects GABA receptors, the vagus nerve system alters the quantity of GABA receptors produced in the brain. This possibly increases the GABA production capacity (108). In addition to short-chain fatty acids and GABA, another potential mechanism linking prebiotics to stress and sleep regulation involves the serotonin pathway. Serotonin, a key neurotransmitter in mood regulation and sleep, is primarily synthesized from tryptophan, an essential amino acid. Approximately 90% of serotonin is produced in the gut, and its synthesis is influenced by gut microbiota composition (109, 110). Certain gut bacteria, including *Bifidobacterium* species, have been shown to modulate tryptophan metabolism, leading to increased serotonin availability in the periphery and potentially affecting central nervous system function (111). In other words, increasing *Bifidobacterium* through prebiotic consumption may be associated with the activation of the serotonin pathway (89). Nevertheless, the gut microbiota likely regulates the brain through multiple complex pathways. However, this study did not measure the metabolites produced by the gut bacteria. Hence, the primary pathways underlying microbial activity may be identified in future studies by measuring the metabolites in the feces and blood and analyzing the correlation between them.

This study has several limitations. First, the diets of the participants were not completely controlled. In this study, we implemented measures such as excluding the individuals who consumed an excessive amount of alcohol or probiotic supplements to mitigate the effects of their usual diets to the highest possible extent; however, their diets were not completely standardized. Furthermore, although the exclusion criteria ruled out individuals taking probiotic preparations or prebiotic supplements, it did not exclude those taking tryptophan or serotonin supplements. Therefore, their usual diet could have affected the study outcomes. Additionally, we administered a food frequency questionnaire to check for major changes in diet before and after the test. However, these surveys were self-reported; hence, the possibility of errors or self-efficacy in the meal data cannot be discounted. Furthermore, because adherence to the test meals was verified solely through self-reported food diaries, the possibility of errors in the dietary data cannot be entirely ruled out. Although the participants were instructed to maintain consistent eating times to the best of their abilities, we were unable to control it. The circadian rhythm of the gut microbiota is linked to the timing of the eating and fasting periods (112, 113). Thus, changes in eating times, such as eating dinner later than usual or shortening the time period between late dinner and breakfast, may possibility exert an effect on the gut microbiota. Furthermore, since there were no specific guidelines regarding the timing of granola consumption, the timing of intake may have had a considerable impact. Second, the time course for the pre-period was increased to 6 weeks because the careful selection of participants took longer than anticipated owing to a large number of volunteers and limited number of participants with Fitbits. Hence, we decided to extend the screening period. Consequently, sleep and stress levels may have varied during these 6 weeks. In fact, several people scored <11 on the Epworth Sleepiness Scale at the pre-screening stage but scored ≥11 at the start of the experiment. Additionally, seasonal variations occur in sleep time and cortisol levels (114, 115). Hence, similar studies must be conducted during other seasons to validate these results. Third, this study was designed as a single-group before-and-after comparison study; therefore, no control group was established. Hence, we could not completely eliminate the possible placebo effects or psychological expectations. A future randomized double-blind study is warranted to confirm the reliability of the results. Finally, unmeasured and uncontrolled confounding factors need to be considered. Social background factors such as economic and marital status could be confounding factors. Additionally, the study participants showed a certain inclination toward a healthy lifestyle. Hence, they may have implemented certain approaches to sleep or stress to improve their lives, and these may have acted as potential biases. Furthermore, mental disorders were not directly assessed through clinical diagnoses or structured interviews. Although we confirmed that none of the participants were taking medications typically prescribed for mental disorders, the possibility that some participants had undiagnosed conditions, such as anxiety or depression, cannot be ruled out. These conditions may have influenced their sleep patterns and responses to the intervention. Therefore, the results of this study should be interpreted within this social context and with considerations made for potential biases.

In summary, we administered the Brief Job Stress Questionnaire to select participants who were experiencing high levels of stress and insomnia for this study requiring the consumption of prebiotics-containing granola for 8 weeks. The results showed improvement in subjective insomnia, initiation and maintenance of sleep, and stress responses. Additionally, the relative abundance of Actinobacteria and *Bifidobacterium* increased, while Bacteriota and *Bacteroides* decreased. Based on these results and multiple regression analysis result, we suggest that increase in *Bifidobacterium* abundance may be associated with improved sleep conditions and stress responses.

## Data Availability

The original contributions presented in the study are included in the article/supplementary material, further inquiries can be directed to the corresponding author/s.

## Supplementary material

The Supplementary material for this article can be found online at: https://www.frontiersin.org/articles/10.3389/fnut.2025.1551313/full#supplementary-material and https://dx.doi.org/10.6084/m9.figshare.28561178

SUPPLEMENTARY FIGURE 1 Flow diagram of the participants. A flow diagram showing the flow of the participants in this study and the criteria for each step.

SUPPLEMENTARY FIGURE 2 Heatmap of the correlation between sleep parameters obtained from EEG measurement and subjective sleep quality from the answers to each questionnaire. Heatmap of the correlation between N1, non-REM sleep stage 1 time; N2, non-REM sleep stage 2 time; N3, non-REM sleep stage 3 time; REM, REM sleep time; TST, Total sleep time; WASO, Wake time after sleep onset; REM ratio, REM sleep ratio; SOL, Sleep onset Latency; AIS, Athens Insomnia Scale; ESS, Epworth Sleepness Scale; and OSA-MA (Factor 1, sleepiness on rising; Factor 2, initiation and maintenance of sleep; Factor 3, frequent dreaming; Factor 4, refreshing; Factor 5, sleep length). The numbers in the diagram represent the correlation coefficient, which was calculated using Spearman’s correlation coefficient (*n* = 27; male *n* = 12; female *n* = 15).

SUPPLEMENTARY FIGURE 3 There is no significant difference in the defecation frequency and the Bristol stool form. **(A)** Defection frequency per week, **(B)** Bristol stool form. The form of the stools is expressed by the following scores from type 1 to 7 (Type 1, separate hard lumps; Type 2, Lumpy and sausage like; Type 3, A sausage shape with cracks in the surface; Type 4, Like a smooth, soft sausage or snake; Type 5, Soft blobs with clear-cut edges; Type 6, Mushy consistency with ragged edges; Type 7, Liquid consistency with no solid pieces). All value are represented as mean ± SEM (*n* = 27; male *n* = 12, female *n* = 15).
