# Supplementary material for: Granola consumption with multiple prebiotics in Japanese participants increases Bifidobacterium abundance and improves stress and subjective sleepiness
Source: Front Nutr. 2025 Mar 20;12:1551313. doi: 10.3389/fnut.2025.1551313 (PMC11965129; doi:10.3389/fnut.2025.1551313)
Supplement: SUPPLEMENTARY FIGURE 1 — Flow diagram of the participants. A flow diagram showing the flow of the participants in this study and the criteria for each step. [file Presentation_1.pptx]

## Slide 1
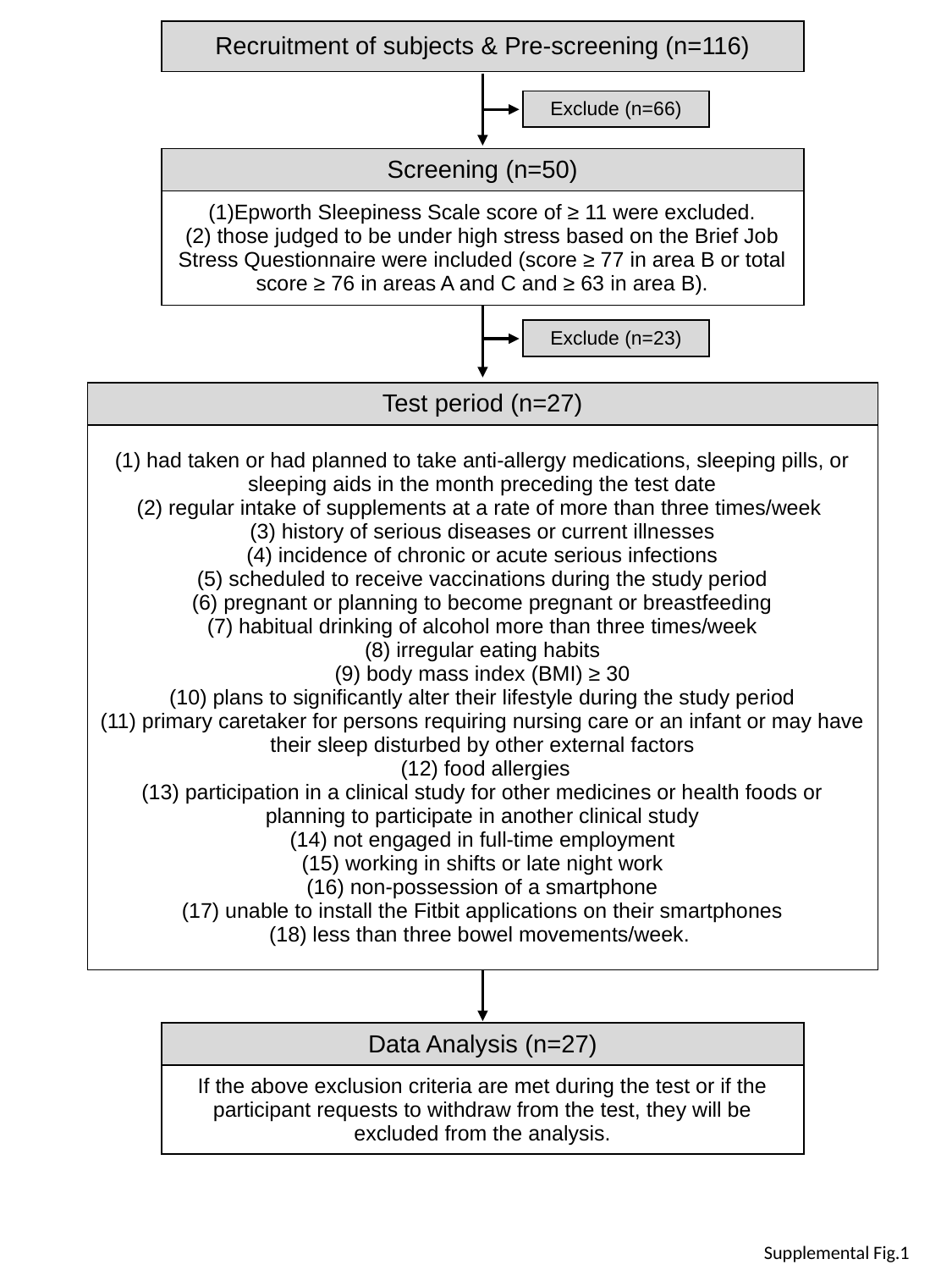

| Recruitment of subjects & Pre-screening (n=116) |
| --- |
| Exclude (n=66) |
| --- |
| Screening (n=50) |
| --- |
| (1)Epworth Sleepiness Scale score of ≥ 11 were excluded. (2) those judged to be under high stress based on the Brief Job Stress Questionnaire were included (score ≥ 77 in area B or total score ≥ 76 in areas A and C and ≥ 63 in area B). |
| Exclude (n=23) |
| --- |
| Test period (n=27) |
| --- |
| (1) had taken or had planned to take anti-allergy medications, sleeping pills, or sleeping aids in the month preceding the test date (2) regular intake of supplements at a rate of more than three times/week (3) history of serious diseases or current illnesses (4) incidence of chronic or acute serious infections (5) scheduled to receive vaccinations during the study period (6) pregnant or planning to become pregnant or breastfeeding (7) habitual drinking of alcohol more than three times/week (8) irregular eating habits (9) body mass index (BMI) ≥ 30 (10) plans to significantly alter their lifestyle during the study period (11) primary caretaker for persons requiring nursing care or an infant or may have their sleep disturbed by other external factors (12) food allergies (13) participation in a clinical study for other medicines or health foods or planning to participate in another clinical study (14) not engaged in full-time employment (15) working in shifts or late night work (16) non-possession of a smartphone (17) unable to install the Fitbit applications on their smartphones (18) less than three bowel movements/week. |
| Data Analysis (n=27) |
| --- |
| If the above exclusion criteria are met during the test or if the participant requests to withdraw from the test, they will be excluded from the analysis. |
Supplemental Fig.1

## Slide 2
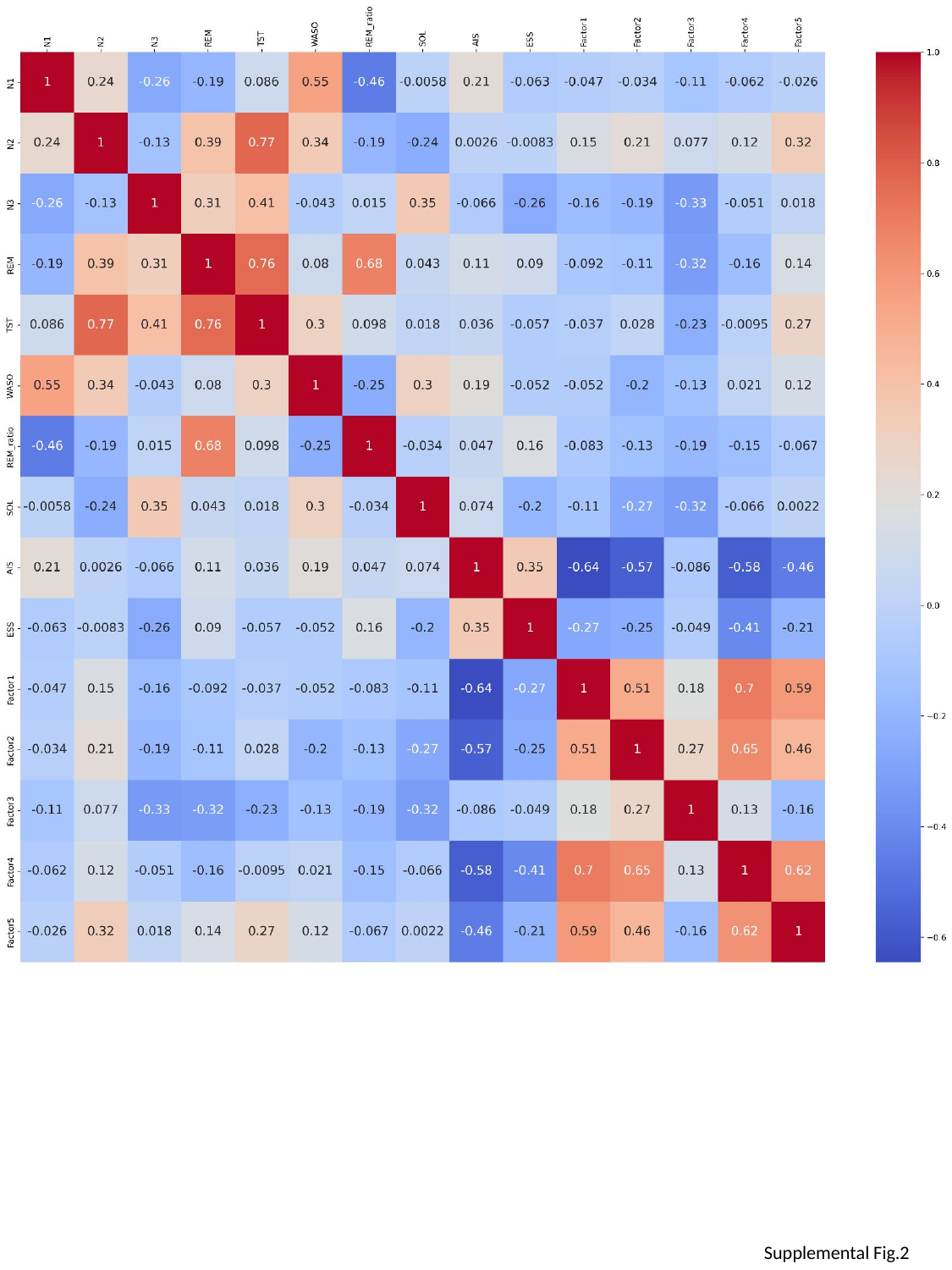

Supplemental Fig.2

## Slide 3
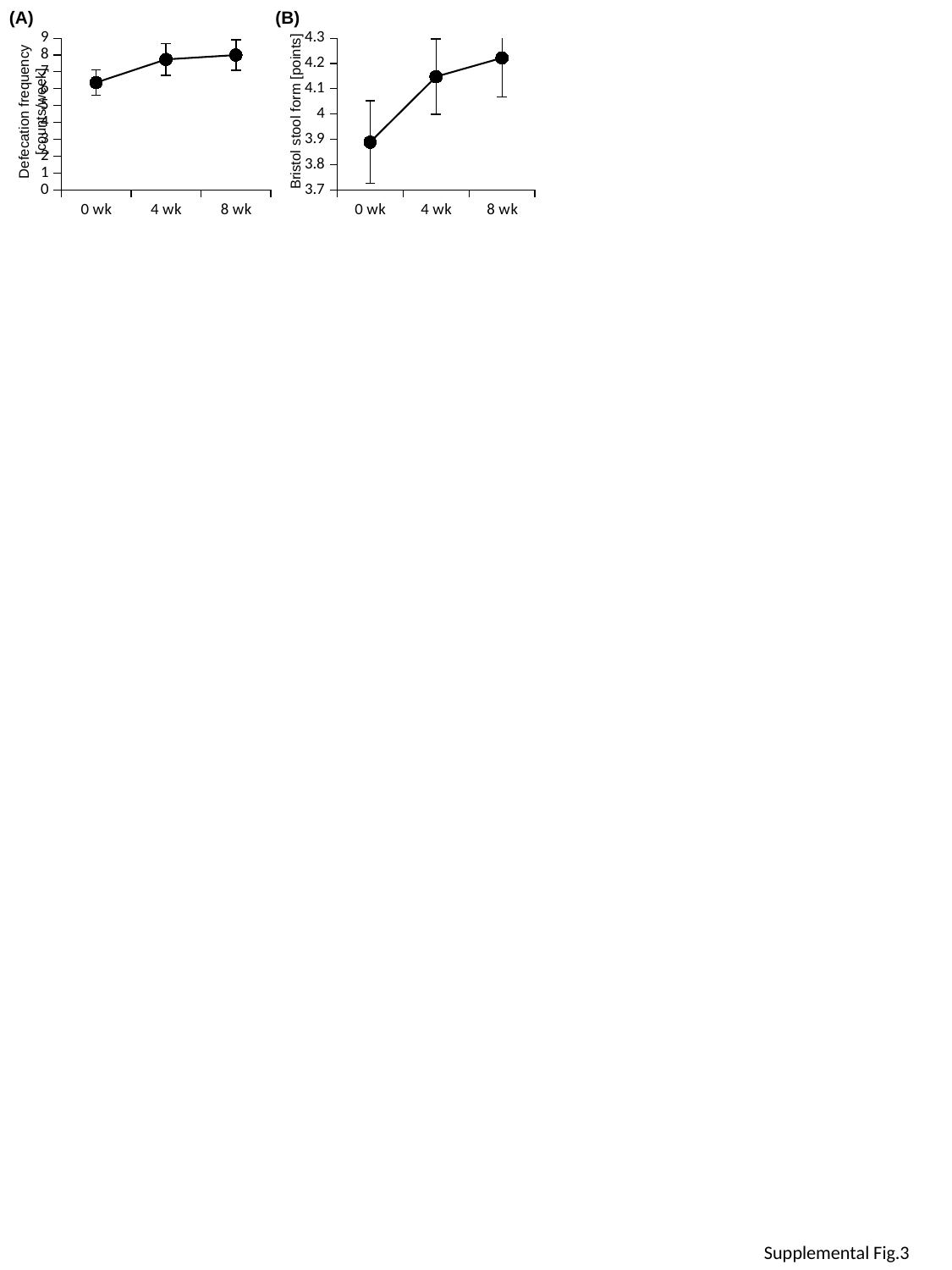

(A)
(B)
### Chart
| Category | 排便回数 |
|---|---|
| 0 wk | 6.37037037037037 |
| 4 wk | 7.7407407407407405 |
| 8 wk | 8.0 |
### Chart
| Category | 便性状 |
|---|---|
| 0 wk | 3.888888888888889 |
| 4 wk | 4.148148148148148 |
| 8 wk | 4.222222222222222 |Defecation frequency [counts/week]
Bristol stool form [points]
Supplemental Fig.3
